# Energy Dynamics of Long-Wave Low-Amplitude Disturbances in an Anharmonic One-Dimensional Lattice

**DOI:** 10.3390/ma18225224

**Published:** 2025-11-18

**Authors:** Stepan Shcherbinin, Julia Baimova, Anton Krivtsov

**Affiliations:** 1 Higher School of Theoretical Mechanics and Mathematical Physics, Peter the Great Saint Petersburg Polytechnical University, 195251 Saint Petersburg, Russia; 2 Institute for Problems in Mechanical Engineering RAS, 199178 Saint Petersburg, Russia; 3 Laboratory of Wave Processes, Yaroslav-the-Wise Novgorod State University, 173003 Veliky Novgorod, Russia; 4 Institute for Metals Superplasticity Problems of the Russian Academy of Sciences, Khalturina 39, 450001 Ufa, Russia

**Keywords:** energy transfer, solitons, anharmonic lattice

## Abstract

We present analytical investigations of evolution of localized disturbances during their propagation in an infinite monoatomic nonlinear one-dimensional lattice, specifically the α-Fermi-Pasta-Ulam (FPU) chain. We focus on two key disturbance characteristics: the position of the energy center and the energy radius. Restricting our analysis to long-wave low-amplitude disturbances, we investigate the dynamics in the α-FPU chain and its two continuous versions described by the Boussinesq and Korteweg–de Vries (KdV) equations. Utilizing the energy dynamics approach and leveraging the known property of the KdV equation that any localized disturbance eventually decomposes into a set of non-interacting solitons and a dispersive oscillatory tail, we establish a similarity between the behavior of the disturbance in the linear chain and the nonlinear chain under consideration. Namely, at large time scales, the disturbance energy center propagates and the energy radius increases linearly in time, meaning dispersion also occurs at a constant velocity, analogous to the linear case. It was also found that, prior to its decomposition into non-interacting components, a disturbance in the KdV equation generally evolves as if subjected to an effective force from the medium. Furthermore, for two reduced versions of the KdV equation—one lacking the dispersive term and the other lacking the nonlinear term—the energy center of any disturbance moves with constant velocity. These results generalize the behavior observed in harmonic chains to weakly nonlinear systems and provide a unified framework for understanding energy transport.

## 1. Introduction

The study of wave energy transfer processes plays a key role for understanding phenomena in various fields of science and technology [1,2,3,4,5]. At the macroscale, consideration of such processes can provide an important information about geological structures [5]. At the micro and nanoscale, the wave processes are tidily connected with nondiffusive ballistic and anomalous processes of heat energy transfer [6,7,8,9,10]. Such phenomena, along with surprisingly high thermal conductivity of polymers and nanocomposites [11,12,13], can be of considerable practical significance. Their research addresses the critical challenge of efficient heat transfer in computer processors and diverse energy systems. The theory of the energy transfer at the nanoscale has become very pronounced with the development of nanotechnologies and nanoscale devises [14,15]. Recent studies have shown that the Fourier law is not satisfied at the micro- and nanoscale, and non-diffusive ballistic and anomalous thermal energy transfer are observed [16,17,18]. Our scientific group has carried out studies of energy transfer processes in miscellaneous discrete low-dimensional systems, such as one-dimensional monoatomic harmonic lattices [19,20,21,22,23], harmonic chain with defects [24,25], metamaterials [26,27] and two-dimensional lattices [28,29]. These studies, together with some earlier ideas [30,31], played a motivating role in the development of the energy dynamics approach [32,33,34], allowing unified consideration of energy transfer processes of different nature. From one hand, this approach is grounded in the well-established analogy between mass and energy transfer processes [35]. From the other hand, it shows, in a sense, an idea of the kinetic description of heat transfer, where phonons (qausiparticles) are responsible for energy transfer [4,22,36].

The quasiparticle-based kinetic formalism provides a considerable simplification over descriptions relying on the full set of lattice dynamics equations. The basis of the kinetic description is the use of the Boltzmann equation for energy-transferring quasiparticles [37,38]. This equation can be both investigated numerically [39,40] as well as used to obtain heat propagation equations [41,42,43]. This approach is effectively used to study thermal transport at low temperatures [44], at the nanoscale [36,45], in low-dimensional materials [10,46,47] and so on. Energy dispersion, crystal anharmonicity, and lattice inhomogeneity influence the size and shape of the energy disturbances associated with quasiparticles involved in energy transfer. In a dispersive media, such energy disturbances will show a continuous size increase, which results in a finite «lifetime» [22]. Consequently, the physical mechanism of energy transfer by quasiparticles is not yet fully clear. The proposed approach of energy dynamics is developed, in part, to address the unclear effects and at the same time to achieve a simplicity of description similar to the kinetic approach.

The idea of energy dynamics can be effectively used to describe different physical phenomena, which opens new opportunities in the understanding of particular mechanisms underlying energy transfer [21]. For example, the descriptions of processes in optics [48] and electrodynamics (high-frequency wave packets, photons), heat and energy transfer in solid-state physics (phonons and other quasiparticles) [33,34], wave-particle duality in quantum mechanics [25], seismic wave propagation, excitons energy transport in semiconductors. For example, exciton energy transport in semiconductors [49,50], where localized excitonic states or wave packets behave analogously to the localized energy disturbances which can be described with energy dynamics. In such systems, strain gradients, moiré potentials, or dielectric anisotropies can act as effective nonlinearities within the medium, which rises the problem of nonlinearity effects on energy propagation.

Despite the idea of energy dynamics being originally developed for anharmonic systems, subsequently its application was largely used for harmonic systems such as the Hooke chain (The Hooke chain is a one-dimensional system of equal particles, where the nearest neighbors are connected by equal linear springs [32]), inhomogeneous harmonic chains [32,34] and many-dimensional harmonic lattices [33]. For example, finite-energy disturbance in the Hooke chain propagates with the constant velocity and a velocity of its dispersion is also constant [32]. The main goal of this study is to analyze the dynamics of a similar disturbance in the simplest anharmonic model and to clarify whether such velocities can be constant in a nonlinear case. As our analysis has shown, these velocities become constant on large time scales.

We focused on the α-FPU chain [51] because it represents the simplest nonlinear version of series expansion for general-type interparticle potential like, for example, Lennard-Jones [52] and Morse [53] potentials. We also chose a long-wave low-amplitude limit to use the KdV (Korteweg–De Vries) approximation and existing comprehensive theory of the KdV solitons (see, for example, books [54,55,56]).

In the present work, the consept and key equations for the α-FPU chain are developed. The conventional continulization technique is applied to continuous versions of the α-FPU system: the Boussinesq equation and the KdV equation. The approach of energy dynamics is applied to describe motion and dispersion of a disturbance in the α-FPU chain. The obtained results are of high importance for the understanding of the energy transfer in the crystals.

## 2. Energy Dynamics in Discrete System

### 2.1. Basic Equations

An infinite one-dimensional monoatomic crystal with interactions between the nearest neighbors only is analyzed. Equations of motion for such a mechanical system reads:

(1) $$m{\ddot{u}}_{n}=F\left(u_{n}-u_{n-1}\right)-F\left(u_{n+1}-u_{n}\right),\;n\in Z,$$

where dot stands for time derivative, $u_{n}$ denotes the displacement of *n*-th particle from the equilibrium position, *t* is time, *m* is the mass of each particle, Z is a set of all integers, and F(…) represents the force acting to *n*-th particle from one of its nearest neighbors. We restrict ourselves to consideration of potential forces only

(2) $$F\left(y\right)=-\frac{\;d\Pi (y)}{\;dy},$$

where Π(y) is a pair interaction potential. It can be, for example, widely used for Lennard–Jones [52] or Morse [53] potentials.

The energy $E_{n}$ of *n*-th particle can be represented as a sum of the kinetic and the potential energies, $K_{n}$ and $\Pi_{n}$, respectively. The kinetic energy of *n*-th particle reads

(3) $$K_{n}=\frac{mv_{n}^{2}}{2},$$

where $v_{n}={\dot{u}}_{n}$ is the velocity of *n*-th particle. Potential energy of *n*-th particle can be written as

(4) $$\Pi_{n}=\frac{1}{2}\Pi \left(u_{n+1}-u_{n}\right)+\frac{1}{2}\Pi \left(u_{n}-u_{n-1}\right),$$

where $\Pi (u_{n+1}-u_{n})$ represents the energy associated with the bond between particles *n* and n+1, while $\Pi (u_{n}-u_{n-1})$ is the energy associated with the bond between particles n−1 and *n*. So, the local energy associated with a single *n*-th particle can be written as follows:

(5) $$E_{n}=\frac{mv_{n}^{2}}{2}+\frac{1}{2}\Pi \left(u_{n+1}-u_{n}\right)+\frac{1}{2}\Pi \left(u_{n}-u_{n-1}\right),$$

and the global energy of the system under consideration reads

(6) $$E=\frac{1}{2}\sum_{n\in Z}E_{n}.$$

Time-differentiation of the Equation (5) with subsequent substitution of derivative ${\dot{v}}_{n}={\ddot{u}}_{n}$ from the Equation (1) gives the equation for local energy balance:

(7) $$\begin{array}{c} {\dot{E}}_{n}=N_{n-\frac{1}{2}}-N_{n+\frac{1}{2}}, \end{array}$$

where

(8) $$N_{n}=\frac{1}{2}\left(v_{n+\frac{1}{2}}+v_{n-\frac{1}{2}}\right)\;F\left(u_{n+\frac{1}{2}}-u_{n-\frac{1}{2}}\right),\;n\in Z^{\prime }=Z+\frac{1}{2}$$

is the power of energy transfer from particle $n+\frac{1}{2}$ to particle $n-\frac{1}{2}$; symbol $Z^{\prime }$ stands for a set of all half-odd-integers. This quantity is related to the local energy flux $H_{n}$ as follows:

(9) $$H_{n}=aN_{n},$$

where *a* represents the lattice step. According to (7), the sum of ${\dot{E}}_{n}$ over all chain particles gives zero; therefore, the global energy is conserved: E=const. Finally, we introduce the global energy flux:

(10) $$H=\sum_{n\in Z^{\prime }}H_{n}=a\sum_{n\in Z^{\prime }}N_{n},$$

which represents the quantity of energy transfer in the system.

### 2.2. Energy Center

Let us consider a finite-energy disturbance using the energy dynamics [32] approach. This approach is based on two concepts: *carrier* and *phantom*. A carrier is a medium that enables energy transfer. A phantom is a virtual body of matter whose mass distribution is equivalent to the energy distribution in the carrier. Considering the propagation of a disturbance with finite energy in some system, one can introduce the phantom as an effective body of mass that describes the behavior of the energy disturbance in the carrier. The carrier constitutes a substance, a field, or any container where energy transfer occurred. Similarly to the conventional description of mass distribution and transfer, the moments of the energy distribution, as well as the derivatives of these moments, which characterize energy transfer and dispersion can be introduced.

Following [32], we introduce the first energy moment as

(11) $$M=\sum_{n\in Z}naE_{n}.$$

Time-differentiation of the first energy moment (11) using the local energy balance Equations (7) and (9) gives

(12) $$\dot{M}=H$$

—changing rate for the first energy moment is equal to the global energy flux (10). Further differentiation gives

(13) $$\ddot{M}=\dot{H}=\Phi ,$$

where

(14) $$\Phi =\frac{a}{2}\sum_{n\in Z^{\prime }}\left(v_{n+\frac{1}{2}}^{2}-v_{n-\frac{1}{2}}^{2}\right)\;F^{\prime }\left(u_{n+\frac{1}{2}}-u_{n-\frac{1}{2}}\right)$$

is the supply of the global energy flux. This quantity is a force analogue for the energy transfer process. The prime in (14) denotes the derivative of a function (the force function in the considered case) with respect to its argument. Formulas (11)–(14) are derived in [32] for more general cases of an inhomogeneous chain.

Now let us calculate position $x_{c}$ of the *energy center* of the considered disturbance:

(15) $$x_{c}=\frac{M}{E}=a\frac{\sum_{n\in Z}nE_{n}}{\sum_{n\in Z}E_{n}}.$$

The above expression is similar to the standard one for the calculation of the center-of-mass position. Then, from (12) it follows that

(16) $$H=E{\dot{x}}_{c}$$

—the global energy flux satisfies the formula that is similar to the formula for momentum, which is equal to the product of the mass of the system and the center-of-mass velocity.

Next, from (13) we get the energy dynamics equation

(17) $$E{\ddot{x}}_{c}=\Phi$$

—the second Newton’s law analogue for the energy transfer process. Now we can consider a *phantom*, which is a virtual body of matter with mass distribution proportional to the energy distribution in the chain. The mass $\overset{˘}{m}$ of the phantom and the force $\overset{˘}{f}$ acting on the phantom can be defined as

(18) $$\overset{˘}{m}=E/c^{2},\;\overset{˘}{f}=\Phi /c^{2},$$

where *c* is a positive constant, representing some characteristic speed in the system. For the phantom, the center of mass position is the same as the energy center position, $x_{c}$. Then, the Equation (17) takes the form

(19) $$\overset{˘}{m}{\ddot{x}}_{c}=\overset{˘}{f},$$

which is the second Newton’s law for the phantom motion. This interpretation allows us to study the motion of a disturbance in the chain in the same way as we study motion of a massive body in space. Then a technique, which is common for classical mechanics, can be applied to describe the disturbance motion and, consequently, it can be used to analyze the energy transfer in the chain [32].

### 2.3. The α-FPU Chain

**Figure 1:**
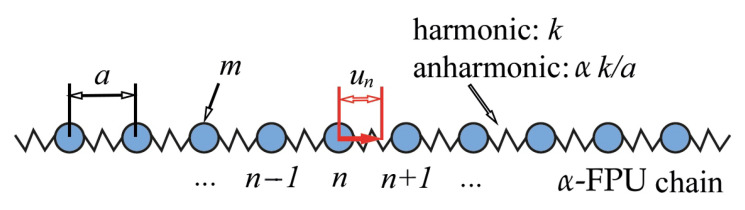
Schematic illustration of one-dimensional monoatomic crystal, where the springs represent couplings between the particles. Here, *m* is the mass of each atom, *a* is the lattice step, *k* is a harmonic bond stiffness, while α is a dimensionless coefficient that characterizes the system’s anharmonicity, $u_{n}$ denotes the displacement of *n*-th particle from the equilibrium position.

The interparticle potential Π(y) of a general type can be rewritten into a Taylor series near y=0, that is, the equilibrium distance between particles:

(20) $$\Pi \left(y\right)=\sum_{j=0}^{\infty }b_{j}y^{j},$$

where $b_{i}$ are some constants. We may set $b_{0}$ = 0 without loss of generality. Constant $b_{0}$ can be set zero without loss of generality. If we accept that for the lattice in equilibrioum, the interparticle forces vanish, then it fulfills that $b_{1}=0$. Constant $b_{2}$ is responsible for the harmonic term of interaction, while $b_{3}$ corresponds to the first anharmonic term. If we restrict ourselves to the case of weak nonlinearity, i.e., the case of small deformations of considered chain, we can neglect in the potential (20) all further terms to obtain the celebrated α-FPU potential:

(21) $$\Pi \left(y\right)=\frac{k}{2}y^{2}+\frac{\alpha k}{3a}y^{3},$$

where *k* is a harmonic bond stiffness, while α is a dimensionless coefficient that characterizes the system’s anharmonicity. Schematic illustration of the considered system one can see in Figure 1.

In this case, the equations of motion (1) read

(22) $$\begin{array}{c} m\frac{\;d^{2}u_{n}}{\;dt^{2}}=k\left(u_{n+1}+u_{n-1}-2u_{n}\right)+\\ +\frac{\alpha k}{a}\left[{\left(u_{n+1}-u_{n}\right)}^{2}-{\left(u_{n}-u_{n-1}\right)}^{2}\right],\;n\in Z. \end{array}$$

Let us note that the more general case, when coefficients $b_{0}$ and $b_{1}$ of the expansion (20) are not zero, gives exactly the same Equations (22).

In case α=0 we have the corresponding harmonic model, that is, the Hooke chain. From Formula (14), it follows that for the Hooke chain the force analog vanishes: Φ=0. For the α-FPU chain from (14) we obtain expression

(23) $$\Phi =-\alpha k\sum_{n\in Z}\left(u_{n+1}-u_{n}\right)\left(v_{n+1}^{2}-v_{n}^{2}\right),$$

that generally is not zero. Therefore, in the α-FPU chain a disturbance moves as if it is affected by an effective force acting from the medium.

## 3. Energy Dynamics in Continuum Limit

**Figure 2:**
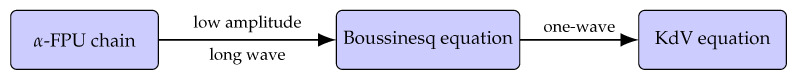
Schematic illustration of the transition from the α-FPU chain to the KdV equation. The dynamics of low-amplitude long-wave disturbances in the α-FPU chain is described by the Boussinesq equation up to second-order terms in the small parameter. The Boussinesq equation, in turn, reduces to the KdV equation under one-wave approximation—i.e., for waves propagating in only one direction.

To analyze the energy dynamics in the α-FPU chain, we examine long-wave small-amplitude disturbances, whose dynamics are governed by the KdV equation. This correspondence was first established in [57], and the derivation of the KdV equation from the α-FPU chain has since been extensively documented in the literature (see, for example, [58]). However, we have chosen to reproduce such a derivation in a form that differs slightly from the conventional one. The difference is that we avoid the explicit introduction of small parameters into the equations. We find this approach to handling small parameters more convenient for analyzing the transformation of energy-related quantities during the transition from the original chain to its continuum counterparts, as it explicitly preserves the original dimensions of all quantities and variables. The transition from the α-FPU to the KdV equation is detailed in the Appendix A, Appendix B and Appendix C. Schematic illustration of this transition one can see in Figure 2.

### 3.1. The Boussinesq Equation

The case of long waves and low amplitudes means that the disturbances under consideration are smooth and generate small deformations. A continuous function $u\left(x,t\right)=u\left(na,t\right)=u_{n}\left(t\right)$ can be used to express displacement of each particle $u_{n}\left(t\right)$ with the small parameters

(24) $$\frac{\partial u}{\partial x}∼\varepsilon_{1},\;\frac{a}{\phi }\frac{\partial \phi }{\partial x}∼\varepsilon_{2}$$

where ϕ is any continuous function to represent the disturbance under consideration, $\varepsilon_{1}$ and $\varepsilon_{2}$ are dimensionless small parameters, responsible for the low-amplitude and the long-wave approximations, respectively. Substituting Taylor expansion of u(x±a,t) into the equations of motion (22) of the α-FPU chain and leaving the first and the second terms taking into account the small parameters $\varepsilon_{1}$ and $\varepsilon_{2}$ we get the equation

(25) $$\rho u_{tt}=Du_{xx}+2\alpha Du_{x}u_{xx}+\frac{a^{2}D}{12}u_{xxxx},$$

where D=ka, ρ=m/a, $u_{x}$ and $u_{t}$ are the corresponding partial derivatives of the function u(x,t). The Equation (25) is the Boussinesq equation. The leading order of the Boussinesq equation (the left side and the first term on the right side) is the standard wave equation, while nonlinear and dispersion terms (the second and the third terms in the right side) provide small corrections. These corrections have the same order in case that

(26) $$\varepsilon_{1}∼\varepsilon ,\;\varepsilon_{2}∼\varepsilon^{\frac{1}{2}},$$

where ε is a dimensionless small parameter (for more details see the Appendix A).

The Equation (25) was presented by J. Boussinesq in his works of 1870s [59,60,61,62,63,64], which was originally used to describe water waves dynamics in a form that is slightly different from (25). The native form of the Boussinesq equation can be obtained in terms of variable $U=u_{x}$ by differentiation of the Equation (25) with respect to the spatial coordinate *x*.

Let us consider the global energy (5) and (6). We use the Taylor expansion of u(x±a,t), consider lattice step *a* as dx, and convert the sum to an integral. Then, leaving the first and the second order terms with respect to the small parameter ε and integrating by parts, we obtain

(27) $$E=\;\int_{-\infty }^{\infty }\;\;\;\epsilon \;dx,\;\epsilon =\frac{\rho u_{t}^{2}}{2}+\frac{D}{2}u_{x}^{2}+\frac{\alpha D}{3}u_{x}^{3}-\frac{a^{2}D}{24}u_{xx}^{2},$$

where ϵ(x,t) is the energy density. The local energy balance Equation (7) transforms now to

(28) $$\epsilon_{t}=-h_{x},$$

where *h* is the local energy flux:

(29) $$h=-D\left[u_{t}u_{x}+\alpha u_{x}^{2}u_{t}+\frac{a^{2}}{12}\left(u_{xxx}u_{t}-u_{xx}u_{xt}\right)\right].$$

It should be noted that the energy density and the corresponding energy flux density are not uniquely defined. Details on the derivation of the Formulas (27) and (29) are provided in the Appendix B.

Analogously to the discrete Formula (11) the first energy moment is

(30) $$M=\int_{-\infty }^{\infty }x\epsilon \;dx,$$

where the sum is replaced with the corresponding integral. The position of the energy center is $x_{c}=M/E$. The time derivative of the first energy moment is

(31) $$\dot{M}=-\int_{-\infty }^{\infty }xh_{x}\;dx=-\int_{-\infty }^{\infty }x\;dh=\int_{-\infty }^{\infty }h\;dx=H,$$

where *H* is the global energy flux. Hence, the global energy flux is related to the energy center velocity in the same manner, as for the discrete system:

(32) $$H=E{\dot{x}}_{c},$$

and the acceleration of the energy center satisfies the same energy dynamics equation

(33) $$E{\ddot{x}}_{c}=\Phi ,$$

where Φ is the continualized force analog acting on the energy disturbance from the medium:

(34) $$\Phi =-2\alpha D\int_{-\infty }^{\infty }u_{t}u_{x}u_{xt}\;dx.$$

This formula can be derived in two ways: by time differentiation of (29) using the equation of motion (25) followed by integration, or by continualization of Formula (23).

### 3.2. The KdV Equation

As it was mentioned, the leading order in the Boussinesq Equation (25) gives the wave equation

(35) $$u_{tt}=c^{2}u_{xx},$$

where

(36) $$c=\sqrt{\frac{D}{\rho }}=\sqrt{\frac{k}{m}}\;a$$

is the speed of long linear waves. The general solution of (35) represented as superposition of two waves of constant shape traveling with speed *c* in opposite directions—this is the well-known d’Alembert solution [65]. Since the nonlinear and dispersion terms in the Boussinesq Equation (25) are the first small correction, it is seen that the Equation (25) should have similar solutions with a slowly changing shape. To study one of these two solutions let us consider a frame of reference moving with speed *c* in the direction of increasing the spacial coordinate. The following coordinates can be associated with this moving frame of reference:

(37) χ=x−ct,τ=t.

Mathematically, these formulas can be considered as a change of variables. For corresponding partial derivatives we have

(38) $$\phi_{t}=\phi_{\tau }-c\phi_{\chi },\;\phi_{x}=\phi_{\chi },$$

where ϕ is an arbitrary function of variables *t*, *x* or variables τ, χ.

The equations of energy dynamics in the moving frame of reference undergo some changes, since the fluxes depend on the velocity of the observer. For the local quantities, this can be illustrated by the energy balance Equation (28) that in terms of parameters τ, χ with the use of (37) takes the from

(39) $$\epsilon_{\tau }-c\epsilon_{\chi }=-h_{\chi }.$$

This equation can be rewritten as

(40) $$\epsilon_{\tau }=-{\tilde{h}}_{\chi },$$

where

(41) $$\tilde{h}=h-c\epsilon$$

is the energy flux density in the moving frame. For global quantities it is clear that the first energy moment in the moving frame is

(42) $$\tilde{M}=\int_{-\infty }^{\infty }\chi \epsilon \;d\chi =\int_{-\infty }^{\infty }(x-ct)\;\epsilon \;dx=M-cEt.$$

Then the global energy flux

(43) $$\tilde{H}={\dot{\tilde{M}}}_{1}=H-cE.$$

Here dot stands for the full time derivative (all global quantities depend on time *t* only). On the other hand it is easy to see that

(44) $$\tilde{H}=\int_{-\infty }^{\infty }\tilde{h}\;d\chi .$$

Further differentiation gives

(45) $$\dot{\tilde{H}}=\dot{H}=\Phi ,$$

where Φ is the force analogue, which is exactly the same for both frames. The energy center coordinate in the moving frame is

(46) $$\chi_{c}=\tilde{M}/E=x_{c}-ct\;\Rightarrow \;{\dot{\chi }}_{c}={\dot{x}}_{c}-c,$$

where dot stands for the time differentiation of the corresponding positions (variables *t* and τ in this case are indistinguishable). Then the energy dynamics equation has the same form in both frames

(47) $$E{\ddot{\chi }}_{c}=E{\ddot{x}}_{c}=\Phi .$$

Thus all the equations of energy dynamics are similar for both frames, but the fluxes and velocities undergo some changes. Note that the observed similarity is due to the fact that the velocity *c* of the moving frame of reference is constant. If not, then we should expect additional terms in the energy dynamics Equation (47), similar to those that appear in the Newtonian dynamics equation for non-inertial reference frames.

All the above formulas in this subsection are exact. The further progress is connected with the possibility of obtaining a simplified approximal equation, which is known as the Korteweg–De Vries (KdV) equation [57]. It was derived for the first time by J. Boussinesq [61,62,63,64] and then described by D. Korteweg and G. de Vries [66]. While the KdV equation was originally derived for unidirectional shallow water waves (in the long-wave, small-amplitude limit), Zabusky and Kruskal [57] first utilized it to describe the α-FPU chain dynamics, as noted previously.

In the moving frame of reference the Boussinesq Equation (25) takes the form

(48) $$\rho u_{\tau \tau }=2\rho cu_{\chi \tau }+2\alpha Du_{\chi }u_{\chi \chi }+\frac{a^{2}D}{12}u_{\chi \chi \chi \chi }.$$

As stated above, we expect a slow change of the shape of a disturbance described by this equation. Therefore we neglect term $u_{\tau \tau }$ and after substitution $D=\rho c^{2}$ we obtain the KdV equation:

(49) $$u_{\chi \tau }+\alpha cu_{\chi }u_{\chi \chi }+\frac{a^{2}c}{24}u_{\chi \chi \chi \chi }=0.$$

The framework in which this equation is obtained is referred to in the literature as one-wave approximation [67,68]. It uses the following asymptotic relations:

(50) $$\frac{\partial u}{\partial \chi }∼\varepsilon ,\;\frac{\partial \phi }{\partial \chi }∼\frac{\phi }{a}\varepsilon^{\frac{1}{2}},\;\frac{d\phi }{d\tau }∼\omega \phi \varepsilon^{\frac{3}{2}},$$

where ϕ=ϕ(τ,χ) is an arbitrary function decribing considered equation and $\omega =\sqrt{\frac{k}{m}}$ (for more details see Appendix C). Let us emphasize that the first two relations in (50) are conditions introduced to derive the Boussinesq equation from the α-FPU chain equations, whereas the third one follows automatically from these conditions within the one-wave approximation.

It can be shown that within one-wave approximation the local energy and flux densities are

(51) $$\begin{array}{c} \epsilon =Du_{\chi }^{2},\\ \tilde{h}=\frac{2\alpha cD}{3}u_{\chi }^{3}+\frac{a^{2}cD}{12}\left(u_{\chi }u_{\chi \chi \chi }-\frac{u_{\chi \chi }^{2}}{2}\right). \end{array}$$

Then the global energy and flux in the moving frame are

(52) $$\begin{array}{c} E=D\int_{-\infty }^{\infty }u_{\chi }^{2}\;d\chi ,\\ \tilde{H}=cD\int_{-\infty }^{\infty }\left(\frac{2\alpha }{3}u_{\chi }^{3}-\frac{a^{2}}{8}u_{\chi \chi }^{2}\right)\;d\chi , \end{array}$$

where integration by parts has been used to simplify the expression. Let us note that both terms in the right side of Formula (52)_2_ have the same order, where the first term is responsible for nonlinearity, the second one for dispersion. Using Formula (52), $\tilde{H}/E$ can be used to find the energy center velocity of a disturbance, that gives

(53) $${\dot{\chi }}_{c}=c\int_{-\infty }^{\infty }\left(\frac{2\alpha }{3}u_{\chi }^{3}-\frac{a^{2}}{8}u_{\chi \chi }^{2}\right)\;d\chi /\int_{-\infty }^{\infty }u_{\chi }^{2}\;d\chi .$$

It can be shown that the force analogue for the KdV equation is

(54) $$\Phi =\alpha \frac{a^{2}c^{2}D}{24}\int_{-\infty }^{\infty }u_{\chi \chi }^{3}\;d\chi .$$

This relatively simple formula can be used to study energy dynamics of solitary waves in the systems under consideration.

### 3.3. The Reduced KdV Equations

Consider the KdV Equation (49). It can be rewritten as

(55) $$w_{\tau }+\alpha cww_{\chi }+\frac{a^{2}c}{24}w_{\chi \chi \chi }=0,$$

where $w=u_{\chi }$ is the deformation of the medium. The second term is to reproduce the nonlinearity, while the third one corresponds to the dispersion, caused by the discreteness of the original chain. The associated parameters are α (the measure of the nonlinearity) and *a* (the lattice step, the measure of the discreteness). As was mentioned before, these both terms are of the same order. However, if for some reasons one of these terms prevails, we obtain the reduced KdV equations.

First, if the nonlinearity prevails and the discreteness is negligible we obtain the equation of a nonlinear wave evolution

(56) $$w_{\tau }+\alpha cww_{\chi }=0,$$

which is the simplest model to study the wave breaking process [2].

Alternatively, if the discreteness prevails and the nonlinearity is negligible we obtain the linearized KdV equation

(57) $$w_{\tau }+\frac{a^{2}c}{24}w_{\chi \chi \chi }=0,$$

which allows us to study in the first approximation the dispersion of long waves in the Hooke chain [69].

Since the force analogue (54) is proportional to both α and *a*, it vanishes for the both reduced KdV equations, (56) and (57). Thus, in both cases we have Φ=0, consequently, the energy flux and the energy center velocity are conserved for the reduced equations. The corresponding constant velocities, according to (53), are

(58) $${\dot{\chi }}_{c}=\frac{2\alpha c}{3}\int_{-\infty }^{\infty }w^{3}\;d\chi /\int_{-\infty }^{\infty }w^{2}\;d\chi$$

—for the nonlinear Equation (56) and

(59) $${\dot{\chi }}_{c}=-\frac{a^{2}c}{8}\int_{-\infty }^{\infty }w_{\chi }^{2}\;d\chi /\int_{-\infty }^{\infty }w^{2}\;d\chi$$

—for the dispersive Equation (57). Here it is used so that the energy in all cases has the same form given by (52)_1_. One can see that the sign of the velocity (58) is defined by the sign of α and *w*. In contrast, the sign of the second velocity (59) is always negative. Let us remind that this velocity is relative; therefore, the negativeness of ${\dot{\chi }}_{c}$ means that the dispersion reduces the absolute velocities of the disturbances in the systems under consideration.

## 4. Energy Dynamics Analysis of the KdV Equation

### 4.1. Energy Transfer

Let us consider the following variables change:

(60) $$\tau =\frac{a}{c\sqrt{24\alpha^{3}}}\zeta ;\;\chi =\frac{a}{\sqrt{24\alpha }}\varkappa ,$$

where θ and ϰ are the dimensionless versions of the corresponding variables. Then we change KdV Equation (55) to the following dimensionless type:

(61) $$w_{\zeta }+ww_{\varkappa }+w_{\varkappa \varkappa \varkappa }=0.$$

This equation has a well-known [70] soliton solution:

(62) $$w=\frac{w_{0}}{\cosh^{2}\left[\gamma (\varkappa -\varkappa_{0}-\nu \zeta )\right]},$$

where $\varkappa_{0}$ is the initial soliton’s position; the soliton’s height $w_{0}$, width γ, and velocity ν are expressed in terms of the arbitrary dimensionless parameter *q*: $w_{0}=3q^{2}$, γ=q/2, $\nu =q^{2}$. Then the soliton solution for (55) will be

(63) $$w=\frac{3q^{2}}{\cosh^{2}\left(\frac{\sqrt{6\alpha }}{a}q\left(\chi -\chi_{0}-q^{2}\alpha c\tau \right)\right)},$$

where $\chi_{0}$ is the initial soliton’s position. Let us calculate now the soliton’s energy

(64) $$E=D\int_{-\infty }^{\infty }w^{2}\;d\chi =\sqrt{\frac{24}{\alpha }}\;q^{3}Da$$

and the first energy moment

(65) $$\tilde{M}=D\int_{-\infty }^{\infty }\chi w^{2}\;d\chi =\left(\chi_{0}+q^{2}\alpha c\tau \right)E.$$

Then the position of the soliton’s energy center and its velocity are

(66) $$\chi_{c}=\frac{M}{E}=\chi_{0}+q^{2}\alpha c\tau ,\;{\dot{\chi }}_{c}=q^{2}\alpha c.$$

Thus, the soliton’s velocity given by the energy dynamics approach is a constant, and the value of this constant coincides with the well-known velocity of the soliton.

**Figure 3:**
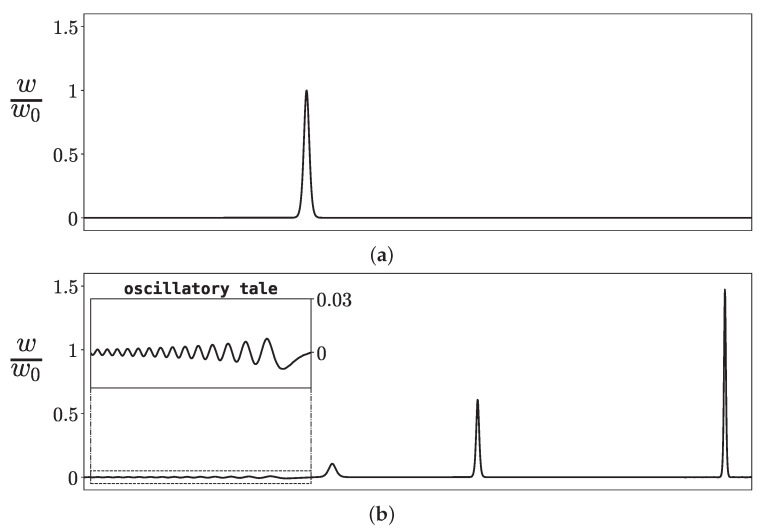
(**a**) Initial disturbance of the form $w_{0}\cosh^{-3}\left(w_{0}\varkappa \right)$ ($w_{0}=0.01$) in the KdV Equation (61). During process of transition, this disturbance transforms into (**b**) a set of solitons and an oscillatory tail. The numeric solution was obtained with the aid of the fast Fourier transform implemented in NumPy [72] Python package (https://www.python.org, 1 November 2025).

We consider now an initial disturbance profile slightly different from the exact soliton (below for brevity $\chi_{0}=0$):

(67) $$w_{0}=\frac{3q_{0}^{2}+\delta }{\cosh^{2}\left(\frac{\sqrt{6\alpha }}{a}q_{0}\chi \right)},$$

where δ is a small parameter, which can be positive or negative. Figure 3 presents the a set of exact solitons moving to the right and an oscillatory dispersive wave, or *oscillatory tale* (for example, [71]), into which the disturbance in the KdV equation after large times transforms.

If such a disturbance is close to an exact soliton, then in general it splits into three parts. The first is a large soliton containing most of the initial energy. The second and the third are the oscillating tail and a small soliton, which contain the remainder of the initial energy (for details see the Appendix D). In other words, the initial disturbance (67) transforms into an exact soliton, losing excess energy. The profile of this exact soliton reads

(68) $$w_{e}=\frac{3q_{e}^{2}}{\cosh^{2}\left(\frac{\sqrt{6\alpha }}{a}q_{e}\chi \right)},\;q_{e}=q_{0}+\frac{2\delta }{9q_{0}},$$

where the expression for $q_{e}$ omits small terms of higher orders by the small parameter δ. This result is obtained with the aid of the inverse scattering transform method [70] (for details see the Appendix D). Now we can calculate the profile velocity at t=0 and t→∞. For the initial profile (67), with the aid of the Formula (53), after the corresponding integration, we have

(69) $$v_{0}={\dot{\chi }}_{c}{|}_{t=0}=\alpha c\left(q_{0}^{2}+\frac{8\delta }{15}\right).$$

For the resulting soliton profile (68), using (66)_2_, one can easily get

(70) $$v_{e}={\dot{\chi }}_{c}{|}_{t\to \infty }=\alpha cq_{e}^{2}=\alpha c\left(q_{0}^{2}+\frac{4\delta }{9}\right).$$

In the Formulas (69) and (70), we again have omitted the higher order terms. Finally

(71) $$v_{0}-v_{e}=\frac{4}{45}\alpha c\delta$$

Thus for δ>0 the initial velocity is greater than the final velocity, and otherwise. Here one can witness the phenomenon of “acceleration” and “deceleration” of the considered disturbance by the carrier. Let us note, that here we compare velocity of the initial disturbance with velocity of the exact soliton into which it transforms; not to be confused with the velocity of the entire disturbance at t→∞, where the oscillatory tale is also presented. The latter is an oscillatory dispersive wave, in which the energy center moves with the negative constant velocity (59).

Finally, let us discuss the evolution of an arbitrary initial disturbance with a finite energy in the KdV equation. As mentioned above, at large time scales any disturbance in the KdV equation transforms into a set of solitons and oscillatory tail. At large times solitons line up one by one, starting with the one of highest amplitude (the most faster) and that with the lowest amplitude (the slowest one) at the end. These solitons are non-interacting and propagate at constant velocities. An oscillatory tail is left behind this set of solitons and at large times it represents the wave packet solution of the linearized KdV equation, which is moving with a constant negative velocity. Therefore, the energy center velocity of any disturbance in the KdV equation, after some transitional process, becomes constant.

### 4.2. Energy Dispersion

Above in the current section we discussed a motion of the disturbance energy center in the carrier. But the spatial distribution of the considered disturbance is of high significance. In a general case, a disturbance disperses, that is it spreads out, gradually reducing its localization. To describe such a dispersion, the second energy moment can be used [32]. For the KdV Equation (49), the raw second energy moment $M^{r}$ can be defined as

(72) $$M^{r}=\int_{-\infty }^{\infty }\chi^{2}\epsilon \;d\chi$$

This expression is similar to the one for calculation of the moment of inertia for a massive body. Additionally, the central second energy moment is

(73) $$M^{c}=\int_{-\infty }^{\infty }{\left(\chi -\chi_{c}\right)}^{2}\epsilon \;d\chi ,$$

where $\chi_{c}$ represents the energy center position. The size of the energy disturbance is obtained from its energy radius ϱ, determined as

(74) $$\varrho =\sqrt{M^{c}/E}.$$

This is an analogue for the inertia radius, used to describe the mass distribution in massive bodies. The central and the raw energy moments are linked by the simple relation [32]:

(75) $$M^{r}=E\chi_{c}^{2}+M^{c},$$

where *E* is the global energy of the considered disturbance.

**Figure 4:**
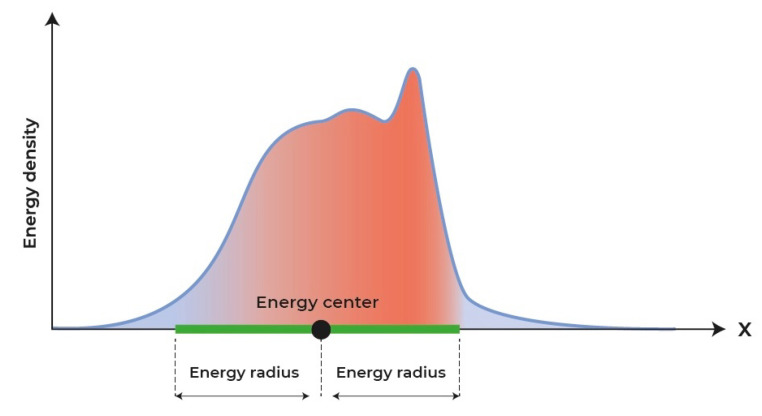
Schematic representation of the energy radius and energy center for a certain disturbance.

For clarity, Figure 4 schematically shows the energy radius together with the energy center for a certain energy disturbance.

In the view of the phantom concept, we can define the raw and the central moments of inertia for the phantom, ${\overset{˘}{\theta }}^{r}$ and ${\overset{˘}{\theta }}^{c}$, similarly to (18):

(76) $${\overset{˘}{\theta }}^{r}=M^{r}/c^{2},\;{\overset{˘}{\theta }}^{c}=M^{c}/c^{2},$$

where *c* is the velocity of long linear waves (36). Then Equation (75) takes the form of the classical Steiner theorem, representing a relation between moments of inertia about parallel axes [73]:

(77) $${\overset{˘}{\theta }}^{r}=\overset{˘}{m}\chi_{c}^{2}+{\overset{˘}{\theta }}^{c},$$

where $\chi_{c}$ plays the role of the distance between an arbitrary axis and the axis through the center of mass of the body, while $\overset{˘}{m}$ is the mass of the phantom defined by the Formula (18).

Now, let us consider a finite energy disturbance of an arbitrary form in the KdV equation. As was already said, at large time scales such a disturbance changes to a set of noninteracting parts (solitons and an oscillatory tail), and each of these parts moves with a constant velocity. So, the right-hand side of the expression (72) at large times breaks down into a sum of the separate terms $M_{i}^{r}$:

(78) $$M^{r}=\sum_{i=0}^{N}M_{i}^{r},\;M_{i}^{r}=E_{i}\chi_{i}^{2}+M_{i}^{c},$$

where i=0 corresponds to the oscillatory tail, i=1,2,…,N corresponds to the sequential solitons, *N* is the number of solitons; $E_{i}$, $\chi_{i}$, $M_{i}^{r}$, and $M_{i}^{c}$ are the global energy, the energy center, the raw moment and the central moment of the corresponding part of the disturbance.

Thus, formula for the central second energy moment of the whole disturbance at large time scales:

(79) $$M^{c}=\sum_{i=0}^{N}\left(E_{i}\chi_{i}^{2}+M_{i}^{c}\right)-E\chi_{c},$$

where *E* and $\chi_{c}$ represent the global energy and the energy center position of the entire disturbance, respectively. Using Formula (73) and the corresponding integration, it is easy to show that the central second energy moment $M_{i}^{c}$ of any soliton (i=1,…,N) is a positive constant. At the same time, the central second energy moment $M_{0}^{c}$ of oscillatory tail is not (see the Appendix E). However, the second time derivative of the second raw moment $M_{0}^{r}$ of the oscillatory tail is a positive constant (see also the Appendix E). The quantities $E_{i}$, ${\dot{\chi }}_{i}$ and *E*, ${\dot{\chi }}_{c}$ are also constant. Therefore, we can find the second time derivative of the central second energy moment of the entire disturbance:

(80) $${\ddot{M}}^{c}=2\sum_{i=1}^{N}E_{i}{\dot{\chi }}_{i}^{2}+{\ddot{M}}_{0}^{r},$$

which turns out to be a constant. Moreover, this constant is positive due to the positiveness of $E_{i}$, $\chi_{i}$ and ${\ddot{M}}_{0}^{r}$.

Thus, we can write down the time dependency for the central second energy moment of a disturbance in the KdV equation at large times in the form

(81) $$M^{c}=C_{1}\tau^{2}+C_{2}\tau +C_{3},$$

where constant $C_{1}={\ddot{M}}^{c}$ while constants $C_{2}$ and $C_{3}$ appear as a result of time integration. Then the time dependency of the energy radius has the form

(82) $$\varrho =\sqrt{(C_{1}\tau^{2}+C_{2}\tau +C_{3})/E}.$$

Consequently, at large times ϱ is a linear function of time:

(83) $$\varrho =v_{\varrho }\tau ,\;v_{\rho }=\sqrt{\frac{C_{1}}{E}}=\sqrt{2\sum_{i=0}^{N}\nu_{i}{\dot{\chi }}_{i}^{2}+\mu },$$

where $v_{\rho }$ is the speed of dispersion, $\nu_{i}=E_{i}/E$ is the constant portion of the global energy that is localized in the *i*-th part of the disturbance, while $\mu =\ddot{M_{0}^{r}}/E$. So, we can conclude that at large time scales any disturbance in the KdV equation disperses at a constant speed $v_{\rho }$.

### 4.3. About the KdV Approximation

Finally, it is worth noting, that numerous works can be found on the KdV approximation of the α-FPU chain for case of long waves and small deformations. So, in [74] obtained that in the FPU chain solitary waves exist, and in work [75], it was established that these waves converge to the soliton solutions of the KdV equation. Additionally, in [76] it was shown that the KdV approximation of an infinite α-FPU chain is valid under some assumptions without restriction to solitary waves. And, finally, in recent work [77] it was established that in small-amplitude limit the KdV approximation for the sufficiently smooth disturbance in an infinite α-FPU chain (Actually, this was done for a more general type of potentials, which include, e.g., the Lennard-Jones and the Toda potentials) is valid for a long time interval. Let us also note that the famous phenomenon of the FPU recurrence [51] can be explained by the fact that the initial disturbance splits out into individual solitons and assemble back multiple times [57]. It is obtained in the finite FPU-chain with periodic boundary conditions. For this case, there are theorems on the validity of the KdV approximation (see, for example, [78] and review [79]) similar to those for the infinite chain.

The foregoing allows us to argue the correctness of using the KdV approximation to describe the dynamics of long-wave low-amplitude disturbances in the nonlinear chain with general interatomic potential of the form (20). Moreover, it can be expected that the data obtained here are also valid for a general nonlinear chain under the mentioned conditions.

## 5. Conclusions

In the present paper, the approach of energy dynamics [32,33,34] was applied for energy transfer description in a nonlinear one-dimensional monoatomic lattice with a pair potential (20) of interparticle interactions. Such a lattice is characterized by nonlinear effects on both long and short spatial scales. We have restricted ourselves to the case of a long-wave low-amplitude localized disturbance in an infinite chain. The dynamics of such a disturbance for a sufficiently long time defined using the KdV equation.

For the considered disturbance, following [32], we have introduced such quantities as the coordinate of the energy center and the energy radius. The former quantity characterizes spacial position of the disturbance while the latter corresponds to its spatial localization. It was shown, that for the reduced KdV equations, preserving either nonliner or dispersive terms, the energy center velocity is constant for any disturbance at any time. For the general KdV equation we have found that a disturbance usually moves as if it were affected by an effective force acting from the medium. In other words, a disturbance moves with an acceleration, which depends on its shape. Herewith, for some certain shapes (e.g., solitons) this acceleration can be zero.

At large time scales, the presented approach allows to describe the dynamics of the considered disturbances in fairly simple terms. It is known from soliton theory that any localized disturbance in the KdV equation, after a certain transient process, splits out into non-interacting parts, which are solitons and an oscillatory tail. We have shown that the energy center of each of these parts moves at a constant velocity. Therefore, at large time scales, the energy center of the entire disturbance also propagates uniformly. Moreover, we have shown that at such large times, the disturbance disperses with the constant velocity. That is, its energy radius increases linearly with time.

Thus, at large time scales, the evolution of a long-wave low-amplitude localized energy disturbance in the considered nonlinear system is similar to that in the linear homogeneous chain [32]. The results of the current study make it possible to deepen the understanding of energy transfer processes and to extend energy dynamics approach for more general nonlinear cases.

In conclusion, it should be emphasized that the obtained results are applicable to any physical context in which the KdV equation provides a valid description of wave evolution. This includes, for example, ion-acoustic waves in plasmas [57,80], pressure waves in liquid–gas mixtures [81], and weakly nonlinear waves in rotating fluids [82]. Heat pulses in crystals can also be described by a KdV-type equation [83]. In such contexts, the spatial region occupied by a disturbance may be interpreted as a discontinuity [84]. Thus, the results provide a general description of the long-time dynamics of the corresponding discontinuity. Moreover, the energetic dynamics approach can yield quantitative estimates of its propagation speed [85].

## Data Availability

The original contributions presented in this study are included in the article. Further inquiries can be directed to the corresponding author.

## Appendix A. Transition from the α-FPU Chain to the Boussinesq Equation: Transformation of the Equations of Motion

Let us start from the dynamic Equation (22) describing the α-FPU chain. Assume we are dealing with a finite energy disturbance, which are smooth and generate small deformations. That is, we consider the case of long-wave low-amplitude disturbances. Then, we can introduce two small parameters:

(A1) $$\frac{\partial u}{\partial x}∼\varepsilon_{1},\;\frac{a}{\phi }\frac{\partial \phi }{\partial x}∼\varepsilon_{2}.$$

The first one, $\varepsilon_{1}$, corresponds to the condition of small deformations while the second one, $\varepsilon_{2}$, corresponds to the condition of smoothness. Function ϕ is any continuous function that can describe the disturbance, such as displacement, deformation, velocity, and so on.

Since the disturbance under consideration is a long-wave one, we can replace displacement of each particle $u_{n}\left(t\right)$ with the value of a continuous function u(x,t) at the corresponding point $u_{n}\left(t\right)=u\left(na,t\right)=u\left(x,t\right)$ and expand latter into the Taylor series:

(A2) $$\begin{array}{c} u_{n\pm 1}\left(t\right)=u\left(x\pm a,t\right)=u\left(x\right)\pm a\frac{\partial }{\partial x}u\left(x\right)+\\ +\frac{a^{2}}{2}\frac{\partial^{2}}{\partial x^{2}}u\left(x\right)\pm \frac{a^{3}}{6}\frac{\partial^{3}}{\partial x^{3}}u\left(x\right)+\frac{a^{4}}{24}\frac{\partial^{4}}{\partial x^{4}}u\left(x\right)+\ldots . \end{array}$$

Substitution of this expansion into (22) gives

(A3) $$\begin{array}{c} mu_{tt}=a^{2}ku_{xx}+\frac{a^{4}k}{12}u_{xxxx}+2\alpha a^{2}ku_{x}u_{xx}+\\ +\frac{\alpha a^{4}k}{3}u_{xx}u_{xxx}+\frac{\alpha a^{4}k}{6}u_{x}u_{xxxx}+\frac{\alpha a^{6}k}{36}u_{xxx}u_{xxxx}+\ldots \end{array}$$

Using conditions (A1), we can estimate the smallness order of terms at the right hand side of the above equation:

(A4) $$\begin{array}{c} a^{2}ku_{xx}∼ku\varepsilon_{2}^{2};\;\frac{a^{4}k}{12}u_{xxxx}∼ku\varepsilon_{2}^{4};\\ 2\alpha a^{2}ku_{x}u_{xx}∼ku\varepsilon_{1}\varepsilon_{2}^{2};\;\frac{\alpha a^{4}k}{3}u_{xx}u_{xxx}∼ku\varepsilon_{1}\varepsilon_{2}^{4};\\ \frac{\alpha a^{4}k}{6}u_{xx}u_{xxx}∼ku\varepsilon_{1}\varepsilon_{2}^{4};\;\frac{\alpha a^{6}k}{36}u_{xxx}u_{xxxx}∼ku\varepsilon_{1}\varepsilon_{2}^{6}. \end{array}$$

Neglecting terms of smallness order $k\varepsilon_{1}\varepsilon_{2}^{4}u$ and higher leads to the Boussinesq equation. Moreover, we can conclude that nonlinear term $2\alpha a^{2}ku_{x}u_{xx}$ and dispersion term $\frac{a^{4}k}{12}u_{xxxx}$ have the same order of smallness only if $\varepsilon_{1}$ and $\varepsilon_{2}$ are connected with relation $\varepsilon_{2}=\varepsilon_{1}^{\frac{1}{2}}$. That is, conditions (A1) takes the form

(A5) $$\frac{\partial u}{\partial x}∼\varepsilon ,\;\frac{a}{\phi }\frac{\partial \phi }{\partial x}∼\varepsilon^{\frac{1}{2}},$$

where $\varepsilon_{1}$ is denoted by ε. Thus, we obtain the Boussinesq equation:

(A6) $$\rho u_{tt}=Du_{xx}+2\alpha Du_{x}u_{xx}+a^{2}D\frac{u_{xxxx}}{12},$$

where D=ka and $\rho =\frac{m}{a}$. Let us emphasize that under conditions (A5), right hand side of the Boussinesq equation takes into account terms of the leading order ($Du_{xx}$) as well as of the first smallness order ($\alpha Du_{x}u_{xx}$ and $a^{2}Du_{xxxx}$).

## Appendix B. Transition from the α-FPU Chain to the Boussinesq Equation: The Energy and the Energy Flux Densities

Let us start from the expression for the local energy of the α-FPU chain

(A7) $$\begin{array}{c} E_{n}=\frac{1}{2}\left(mv_{n}^{2}+\frac{k}{2}{\left(u_{n+1}-u_{n}\right)}^{2}+\frac{k}{2}{\left(u_{n}-u_{n-1}\right)}^{2}+\right.\\ \left.+\frac{\alpha k}{3a}{\left(u_{n+1}-u_{n}\right)}^{3}+\frac{\alpha k}{3a}{\left(u_{n}-u_{n-1}\right)}^{3}\right) \end{array}$$

In continuum limit this expression represents an infinitesimal small portion of energy dE which can be expressed as follows

(A8) $$E_{n}=\;dE=\epsilon \;dx,$$

where ϵ represents energy density while dx is the lattice step *a*.

By substituting the Taylor expansion (A2) into the Equation (A7) for the local energy, taking into account the Formula (A8), and retaining terms up to the first order in smallness, one obtains the following equation for the energy density corresponding to the Boussinesq equation:

(A9) $$\epsilon =\frac{\rho u_{t}^{2}}{2}+\frac{D}{2}u_{x}^{2}+\frac{\alpha D}{3}u_{x}^{3}+\frac{a^{2}D}{8}u_{xx}^{2}+\frac{a^{2}D}{6}u_{x}u_{xxx}.$$

Differentiating this energy density with respect to time and substituting $u_{tt}$ from (A6) leads to the following local conservation law:

(A10) $$\epsilon_{t}=-h_{x},$$

where

(A11) h=−D112utux+αux2ut+a212(2uxuxxt+uxxuxt+uxxxut).

On the other hand, we can consider the local energy flux for the α-FPU chain based on the Formulas (8) and (9), which for the case of integer *n* (n∈Z) read:

Hn=−a2112kvn(un+1−un−1)+αka(vn+1+vn)(un+1−un)2.

Substituting the Taylor expansion (A2) into the above equation and keeping terms of the leading order and the first order of smallness, one can obtain:

(A12) $$h=-D\left[u_{t}u_{x}+\alpha u_{x}^{2}u_{t}+\frac{a^{2}}{6}u_{xxx}u_{t}\right].$$

The energy flux densities in the forms (A11) and (A12) give one and the same expression for the global energy flux:

$$H=\int_{-\infty }^{\infty }h\;dx.$$

This is easy to see using integration by parts and taking into account that $u_{x}\to 0$ as |x|→∞.

Thus, the energy flux density for the Boussinesq equation can be defined in multiple ways. The similar statement is true for the energy density. So, if we define it as follows

(A13) $$\epsilon =\frac{\rho u_{t}^{2}}{2}+\frac{D}{2}u_{x}^{2}+\frac{\alpha D}{3}u_{x}^{3}-\frac{a^{2}D}{24}u_{xx}^{2},$$

this expression will give exactly the same global energy as (A9), since difference of (A9) and (A13) is a complete spatial derivative. Differentiation of energy density (A13) with respect to time and substitution $u_{tt}$ from (A6) leads to the energy flux density in the form

(A14) h=−D112utux+αux2ut+a212(uxxxut−uxxuxt).

This form of the energy flux density gives the same global energy flux as (A11) and (A12). Thus, there is uncertainty in the definition of a specific expressions for densities of energy and energy flux. We choose corresponding forms (A13) and (A14) because of simplicity of writing the energy density.

Note that, in general, distinct energy densities differing by a total spatial derivative—and thus resulting in the same global energy—can correspond to energy flux densities that yield different global energy fluxes. However, in our case, as shown above, this does not occur.

## Appendix C. Transition from the Boussinesq Equation to the KdV Equation

To make a transition to the KdV equation let us change variables in the following manner:

(A15) χ=x−ct,τ=t.

where $c=\sqrt{\frac{k}{m}}a$ represents the speed of linear waves in the considered system. This variables change gives rise to the relations (38). Then second time derivative of *u* can be written as:

(A16) $$u_{tt}=u_{\tau \tau }-2cu_{\tau \chi }+u_{\chi \chi }$$

and the Boussinesq Equation (A6) takes the form

(A17) $$\frac{\rho }{2}u_{\tau \tau }=\rho cu_{\chi \tau }+\alpha Du_{\chi }u_{\chi \chi }+a^{2}\frac{D}{24}u_{\chi \chi \chi \chi }.$$

Since the leading order of the Boussinesq equation corresponds to the standard wave equation, while nonlinear and dispersion terms represent the first small correction, the slow change of the shape of disturbance described by this equation is expected. So, if we neglect term $u_{\tau \tau }$, we obtain the KdV equation:

(A18) $$\rho cu_{\chi \tau }+\alpha Du_{\chi }u_{\chi \chi }+a^{2}\frac{D}{24}u_{\chi \chi \chi \chi }=0.$$

Now we can estimate the smallness order of the neglected term. Rewrite the above equation in the form of the KdV equation:

(A19) $$u_{\chi \tau }=-\alpha \omega au_{\chi }u_{\chi \chi }-\frac{\omega }{24}a^{3}u_{\chi \chi \chi \chi },$$

where $\omega =\sqrt{\frac{k}{m}}$. It is clear from (38) that the conditions (A1) are also valid for derivatives with respect to the new spatial variable χ. That is,

(A20) $$\frac{\partial u}{\partial \chi }∼\varepsilon ,\;{\frac{a}{\phi }\frac{\partial \phi }{\partial \chi }∼\varepsilon }^{\frac{1}{2}}.$$

Then, using the conditions (A20), we can write the following:

(A21) $$u_{\chi \tau }∼\alpha \omega \varepsilon^{\frac{3}{2}}u_{\chi }+\frac{\omega }{24}\varepsilon^{\frac{3}{2}}u_{\chi }∼\omega u_{\chi }\varepsilon^{\frac{3}{2}}.$$

Therefore we can write down the following condition on the derivative with respect to time τ of any function ϕ(χ,τ) describing the considered disturbance:

(A22) $$\frac{d\phi }{d\tau }∼\omega \phi \varepsilon^{\frac{3}{2}}.$$

We emphasize that the condition (A22) represents a consequence of the conditions (A20) in frame of one-wave approximation. Estimation of the smallness orders of the terms in (A17) gives

(A23) $$\begin{array}{c} \frac{\rho }{2}u_{\tau \tau }∼\rho \omega^{2}u\varepsilon^{3};\;\rho cu_{\chi \tau }∼\rho \omega^{2}u\varepsilon^{2};\\ \alpha Du_{\chi }u_{\chi \chi }∼\rho \omega^{2}\varepsilon^{\frac{5}{2}}u;\;a^{2}\frac{D}{24}u_{\chi \chi \chi \chi }∼\rho \omega^{2}\varepsilon^{\frac{5}{2}}u. \end{array}$$

Thus, we can see that term $u_{\tau \tau }$ indeed has a higher order of smallness than other terms, and therefore it can be neglected.

## Appendix D. Initial Disturbance in the Form of the Square of the Hyperbolic Secant in the KdV Equation

Let us present the KdV Equation (61) in the form:

(A24) $$\eta_{\zeta }-6\eta \eta_{\varkappa }+\eta_{\varkappa \varkappa \varkappa }=0,$$

where

(A25) $$\eta =-\frac{w}{6}.$$

It is known from the inverse scattering theory [2,70] that solution of (A24) for the initial disturbance $\eta_{0}\left(\varkappa \right)$ of arbitrary form is uniquely determined by the eigenvalue problem of the Schrodinger equation

(A26) $$\psi_{\varkappa \varkappa }+\left(\lambda -\eta_{0}\left(\varkappa \right)\right)\psi =0.$$

In particular, initial disturbance $\eta_{0}\left(\varkappa \right)$ produces as many solitons as there are discrete eigenvalues of the problem (A26). Moreover, the soliton solution corresponding to the specific eigenvalue $\lambda_{i}=-q_{i}^{2}/4$ has the form:

(A27) $$\eta_{i}=-\frac{q_{i}^{2}}{2\cosh^{2}\left(\frac{q_{i}}{2}\left(\varkappa -\varkappa_{0}\right)-\frac{q_{i}^{3}}{2}\zeta \right)}.$$

Now let us consider the initial disturbance of the form:

(A28) $$\eta_{0}\left(\chi \right)=-\frac{A}{\cosh^{2}\left(\alpha \varkappa \right)},$$

where *A* and α represent arbitrary constants. For such a case, the solution to the problem (A26) is well known, and the discrete spectrum of eigenvalues $q_{i}$ has the form:

(A29) $$q_{i}=\alpha \left[{\left(1+\frac{4A}{\alpha^{2}}\right)}^{\frac{1}{2}}-\left(2i-1\right)\right]>0,\;i=1,2,\ldots .$$

We consider the initial disturbance for the KdV Equation (A24) corresponding to profile (67) for the KdV Equation (55). That is

(A30) $$A=\frac{q_{0}^{2}}{2}+\frac{\delta }{6},\;\alpha =\frac{q_{0}}{2}$$

We remind that δ represents small, positive or negative, parameter. Looking at the Formulas (A29) and (A27), it is easy to see the following. When delta is zero, the considered profile corresponds to an exact soliton. If δ is negative, an exact soliton is formed, and the excess energy remains in an oscillatory tail. If δ is positive, an exact soliton is also formed, but the excess energy remains in an oscillatory tail and another soliton, whose amplitude, velocity and energy are small due to the smallness of δ.

In the last two cases, parameter $q_{e}$ corresponding to the exact soliton containing the main part of the initial energy, according to the Formula (A29), can be written as

(A31) $$q_{e}=\frac{q_{0}}{2}\left(\sqrt{9+\frac{8\delta }{3q_{0}^{2}}}-1\right).$$

Taking into account the smallness of δ as well as the Formulas (60) and (A25), we get the expressions (68).

## Appendix E. Conservation Laws and the First Two Energy Moments for the Linearized KdV Equation

Let us consider the linearized KdV equation in the form:

(A32) $$w_{\tau }+a^{2}\frac{c}{24}w_{\chi \chi \chi }=0,$$

where $w=u_{\chi }$. Zeroing the nonlinear coefficient α in the expressions (51), one can obtain the following forms of the energy density and the energy flux density for the linearized KdV equation:

(A33) $$\epsilon =Dw^{2},\;\tilde{h}=\frac{a^{2}cD}{12}\left(ww_{\chi \chi }-\frac{w_{\chi }^{2}}{2}\right).$$

We emphasize that the energy density of the original KdV equation, being independent of the nonlinear coefficient α, completely coincides with the energy density of the linearized equation. Differentiating with respect to the time τ of (A33)_1_ and substituting $w_{\tau }$ from (A32) leads to the local conservation law:

(A34) $$\epsilon_{\tau }=-{\tilde{h}}_{\chi },$$

where the energy flux density $\tilde{h}$ coincides with that from the formula (A33)_2_. Further, substituting $w_{\tau }$ from (A32) into the τ-derivative of (A33)_2_ leads to the local conservation law for the energy flux density:

(A35) $${\tilde{h}}_{\tau }=-g_{\chi },$$

where quantity *g* can be called the superflux density (following the notation in [32]) and has the form:

(A36) $$g=\frac{a^{4}c^{2}D}{288}\left(\frac{3}{2}w_{\chi \chi }^{2}+ww_{\chi \chi \chi \chi }-2w_{\chi }w_{\chi \chi \chi }\right).$$

The global superflux, which, after integrating by parts the corresponding terms, can be written in the form

(A37) $$G=\int_{-\infty }^{\infty }q\;d\chi =\frac{a^{4}c^{2}D}{64}\int_{-\infty }^{\infty }w_{\chi \chi }^{2}\;d\chi ,$$

represents a conserving quantity. One can prove (A37), differentiating it with respect to the time and substituting $w_{\tau }$ from (A32). Let us note that there is a local conservation law for the superflux density, but it has a sufficient cumbersome form, so we preferred not to present it here.

The constancy of the second time derivative of the raw second energy moment follows from the conservation of the global superflux. Indeed,

(A38) $$\begin{array}{c} {\ddot{M}}^{r}=\int_{-\infty }^{\infty }\chi^{2}\epsilon_{\tau \tau }\;d\chi =-{\left(\int_{-\infty }^{\infty }\chi^{2}{\tilde{h}}_{\chi }\;d\chi \right)}_{\tau }=\\ =2\int_{-\infty }^{\infty }\chi {\tilde{h}}_{\tau }\;d\chi =-2\int_{-\infty }^{\infty }\chi g_{\chi }\;d\chi =2\int_{-\infty }^{\infty }g\;d\chi =2G, \end{array}$$

so we can conclude that quantity ${\ddot{M}}^{r}$ represents a positive constant for any disturbance in the linearized KdV equation.
